# Association between the polymorphisms of CALM1 gene and osteoarthritis risk: a meta-analysis based on observational studies

**DOI:** 10.1042/BSR20181128

**Published:** 2018-10-31

**Authors:** Haoyu Yang, Zhiyong Hu, Chao Zhuang, Ruiping Liu, Yunkun Zhang

**Affiliations:** Department of Orthopedics, The Affiliated Changzhou No.2 People’s Hospital of Nanjing Medical University, Changzhou 213003, China

**Keywords:** CALM1, meta-analysis, OA, polymorphism, systematic review

## Abstract

The existing studies on the association between polymorphisms of *Calmodulin 1* (*CALM1*) gene and the risk of osteoarthritis (OA, a complex multifactorial disease and a major degenerative form of arthritis) in different populations have yielded conflicting findings. Therefore, we conducted a meta-analysis by systematically searching PubMed, Embase, Medline, Cochrane Library and Google Scholar, and assessing this association by calculating pooled odds ratios with 95% confidence intervals. Subgroup analyses stratified by ethnicity, OA type, and genotype were also conducted. Six studies (2752 cases and 3259 controls) involving six single nucleotide polymorphisms were included. Our data suggested that the T allele and genotype TT of the rs12885713 polymorphism, and the C allele of the rs2300496 polymorphism in the *CALM1* gene all increased the risk of OA. The pooled results revealed no significant association between the *CALM1* rs3213718 polymorphism and the risk of OA. Stratification analyses by ethnicity and OA type showed that the rs12885713 polymorphism increased the risk of OA among Asians and in knee OA, respectively. In conclusion, the rs12885713 and rs2300496 polymorphisms of the *CALM1* gene may both increase the risk of OA. Owing to the limitations of the present study, this finding should be further confirmed in future well-designed studies.

## Introduction

Osteoarthritis (OA), the most common form of arthritis, can cause progressive loss of joint function [1]. The distinguishing feature of OA is progressive degradation of articular cartilage accompanied by subsequent joint space narrowing and osteophyte formation at the joint margin, which together lead to chronic joint pain, deformity, and restricted motion [2,3]. OA is a combined result of environmental and genetic factors, which account for nearly 50% of the risk of OA development [4]. Prior genome-wide association studies have [5–7] suggested polymorphisms in some genes may affect OA pathogenesis. Recently, Gao et al. [8] have conducted a meta-analysis indicating that the *SMAD3* gene rs12901499 polymorphism increases the risk of OA among both Asians and Caucasians. Lv et al. [9] have also conducted a meta-analysis indicating that the *ADAM12* gene rs1871054 polymorphism increases knee OA risk in the Asian population, whereas other polymorphisms (rs3740199, rs1044122, or rs1278279) in ADAM12 are not associated with knee OA in any population. Pan et al. [10] have performed an updated meta-analysis suggesting that the C allele and CC genotype of the *GDF5* gene are protective against knee OA susceptibility across different populations. Moreover, single-nucleotide polymorphisms (SNPs) in candidates, such as growth differentiation factor 5 genes, [11] vitamin D receptor, and [12] estrogen receptor-α [13] have been reported to be associated with OA. All the abovementioned SNPs of different genes contribute differently to the risk of OA development through various mechanisms. We hypothesized that candidate gene studies might provide insight into OA development.

Calmodulin (CALM) regulates many Ca^2+^-dependent cellular events involving an interplay among various proteins [14]. Ca^2+^-CALM signaling plays a crucial role in cartilage phenotype maintenance and chondrogenesis. CALM probably has pivotal roles in articular cartilage by maintaining the cartilage phenotype in response to mechanical stimuli in mature chondrocytes [15,16], regulating articular chondrogenesis [17], and modulating the adhesion of chondrocytes to extracellular matrix proteins during cartilage repair. Thus, CALM1 may be involved in OA pathogenesis.

To date, several studies [17–22] have explored the relationship between polymorphisms of the *CALM1* gene, which is located on chromosome 14q32.11, and OA susceptibility. The association between *CALM1* gene SNPs and OA susceptibility may provide new research directions for OA studies. However, the findings of previous studies are conflicting and inconclusive because of clinical heterogeneity, different ethnic populations, and small sample sizes. A single case–control study is underpowered and inconclusive, owing to limited sample sizes. Meta-analysis is used to combine findings based on individual research studies to yield robust conclusions, especially when results from single case–control studies are incomprehensive and conflicting. Thus, to precisely elucidate the genetic roles of *CALM1* gene polymorphisms in OA development, we performed a comprehensive systematic review and meta-analysis to clarify the association between these SNPs and OA risk.

## Materials and methods

This meta-analysis was performed in compliance with PRISMA guideline [23] (not registered, Supplementary Table S1).

### Search strategy

PubMed, Embase, Medline, Cochrane Library, and Google Scholar were systematically searched to identify epidemiological studies published through January 2018 and to retrieve the genetic association studies on OA. The terms ‘Calmodulin’, ‘CALM1’, ‘SNP’, ‘polymorphism’, ‘variant’, ‘osteoarthritis’, and ‘OA’ were used to find all publications reporting *CALM1* gene polymorphisms and OA risk. No language or other restrictions were placed on the search. Full text was obtained if the abstract was insufficient to allow us to include or exclude a study. Furthermore, the reference lists of all the related citations were examined to identify any initially omitted studies.

### Inclusion and exclusion criteria

The inclusion criteria were as follows: (1) case–control study on humans; (2) observational study addressing OA patients and controls; (3) study evaluating the association between *CALM1* gene polymorphisms and susceptibility to OA; and (4) study with sufficient genetic frequency for extraction. The exclusion criteria were as follows: (1) incomplete data; (2) review or case report; and (3) duplicate or overlapping publication. All questionable publications were discussed and addressed by consensus. Three reviewers independently screened the titles and abstracts. In cases of uncertainty regarding any of the above essential information, the full article was retrieved for further scrutiny, or the authors of the individual trials were contacted directly for further information when necessary.

### Data extraction and quality assessment

From each eligible study, two reviewers (Y.H.Y and H.Z.Y) independently extracted the following data: first name of the first author, year of publication, country and ethnicity of study population, type of OA, source of controls (SOC), sample size, genotype method, names of gene polymorphisms, and genotype frequencies in cases and controls. The two reviewers (Y.H.Y and H.Z.Y) independently assessed the methodological quality of the included studies according to the Newcastle–Ottawa Scale (NOS) [24]. The NOS criteria were scored on the basis of three aspects: (1) subject selection, 0–4; (2) comparability of subject, 0–2; and (3) exposure, 0–3. The total NOS scores ranged from 0 (lowest) to 9 (highest). All disagreements were resolved through discussion by consensus or through consultation with the senior reviewer if necessary. Hardy–Weinberg equilibrium (HWE) in controls was tested with Pearson’s χ^2^ test (http://ihg.gsf.de/cgi-bin/hw/hwa1.pl). Studies with scores ≥6 were considered high quality.

### Statistical analysis

Pooled odds ratios (ORs) with 95% confidence intervals (CIs) were calculated to assess the association between *CALM1* gene polymorphisms and OA susceptibility. Owing to a lack of original data for sex and age, crude ORs were calculated. Five genetic models were used: (1) allele model; (2) recessive model; (3) homozygous model; (4) heterozygous model; and (5) dominant model. *P* < 0.05 was considered significant. Heterogeneity across studies was assessed by using the Q statistic with its *P*-value and *I*^2^ statistic [25,26]. If *I*^2^<50% and *P*>0.10, a fixed effects model was used in the calculations [27]; otherwise, a random effects model was applied [28]. Subgroup analyses were carried out on the basis of ethnicity, type of OA, and genotype methods. Potential publication bias was assessed with Egger’s and Begg’s linear regression tests [29]. Sensitivity analysis was performed by omitting each study in turn to determine the effect on the heterogeneity test and evaluate the stability of the overall results. All statistical analyses were conducted in Stata 11.0 (Stata Corporation, College Station, TX, U.S.A.). The significant findings were evaluated by calculating the false-positive report probability (FPRP) [30]. An FPRP threshold of 0.2 and a prior probability of 0.25 were set to detect an OR for a correlation with the tested genotype. FPRP <0.2 implied a significant relationship [31,32].

### Functional prediction

Potential functions of SNPs were extracted from dbSNP (https://www.ncbi.nlm.nih.gov/snp/). We also used bioinformatics databases such as Promo (http://alggen.lsi.upc.es/cgi-bin/promo_v3/promo/promoinit.cgi?dirDB=TF_8.3) and the MirSNP database (http://bioinfo.bjmu.edu.cn/mirsnp/search/) to analyze transcription factor binding sites (TFBS).

## Results

### Characteristics of the included studies

**Figure 1:**
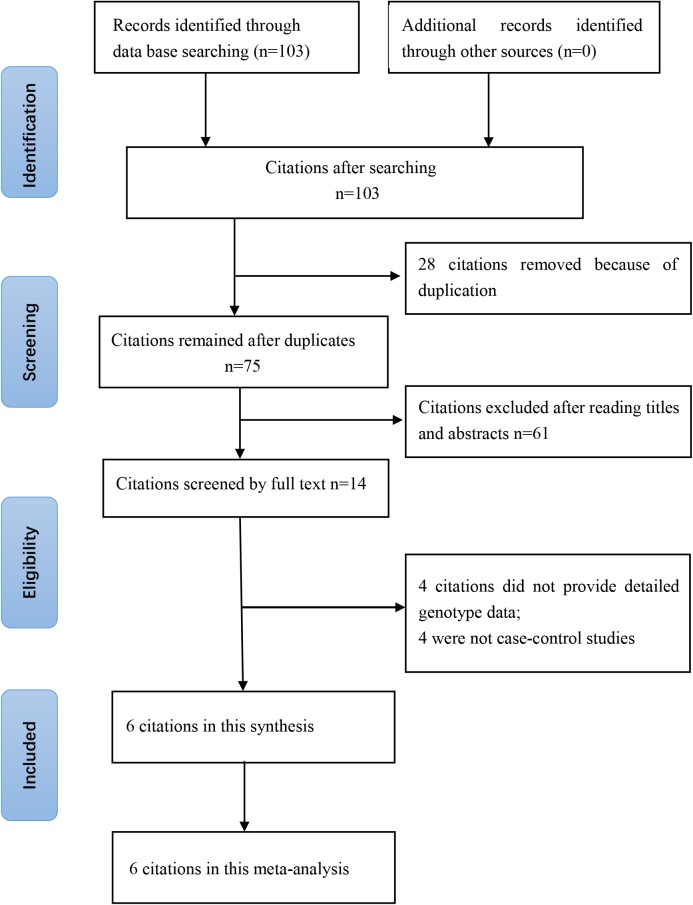
Flowchart of the literature search and selection for the present study

The online search yielded 103 citations, of which 28 duplicates were removed. Then 61 of the 75 remaining citations were excluded after reviewing of titles and abstracts. The remaining 14 citations were sent for full text review, which excluded four citations without detailed genotype data and four non-case–control studies. Finally, six studies (2752 cases and 3259 controls) involving six SNPs were included. The year of publication ranged from 2005 to 2017. The numbers of cases and controls ranged from 183 to 920 and from 193 to 1008, respectively. Two included citations [17,21] studied the association between *CALM1* gene polymorphisms in an Asian population and four included citations [18–20,22] studied Caucasian population. Five of included citations [17–21] studied the association between the rs12885713 polymorphism of the *CALM1* gene and risk of OA. Mishra et al. [19] and Mototani et al. [17] found that the TT genotype of the rs12885713 polymorphism increases the risk for OA, while Shi et al. [21], Poulou et al. [20], and Loughlim et al. [18] did not find a significant correlation between the rs12885713 polymorphism and OA risk. More characteristics of the included citations are shown in Tables 1 and 2. A flowchart of reviews, showing the detailed selection process, is illustrated in Figure 1. The NOS scores ranged from six to seven stars, thus suggesting that the included studies were all of high methodological quality [33–35].

**Table 1:** Characteristics of included studies

Author	Year	Nationality	OA type	Sample size (Female/male)		Age (mean)		Study SNPs	Genotype method	NOS			HWE (*P*-value)
				Case	Control	Case	Control			I	II	III	
Mishra [19]	2017	India	Knee	500 (295/205)	500 (276/224)	F: 55.67	F: 55.52	rs12885713	TaqMan	3	1	3	Y (0.13)
						M: 56.15	M: 54.95	rs3814843	TaqMan	3	1	3	Y (0.59)
								rs2300496	TaqMan	3	1	3	Y (0.11)
Shi [21]	2008	China	Knee	183 (124/59)	210 (142/68)	58.6	57.7	rs12885713	PCR-RFLP	3	1	3	Y (0.73)
Poulou [20]	2008	Greece	Knee	158 (138/20)	193 (137/56)	F: 68.1	F: 60.0	rs12885713	PCR-RFLP	3	1	3	Y (0.13)
						M: 72.4	M: 70.2		PCR-RFLP	3	1	3	
Valdes [22]	2007	U.K.	Knee	603 (305/298)	596 (296/300)	F: 73.5	F: 72.1	rs3213718	PCR-RFLP	3	1	3	Y (as reported)
						M: 72.1	M: 71.8		PCR-RFLP	3	1	3	
Loughlin [18]	2006	U.K.	Hip	920 (547/373)	752 (393/359)	64	69	rs12885713	PCR-RFLP	2	1	3	Y (0.28)
Mototani [17]	2005	Japan	Hip	428 (404/24)	1008 (491/517)	53.7	46.7	rs12885713	TaqMan	3	0	3	Y (0.27)
								rs2300496	TaqMan	3	0	3	Y (0.30)
								rs2300500	TaqMan	3	0	3	Y (0.27)
								rs3213718	TaqMan	3	0	3	Y (0.20)
								rs3179089	TaqMan	3	0	3	Y (0.07)

I, Selection; II, Comparability; III, Exposure. NOS is available from http://www.ohri.ca/programs/clinical_epidemiology/oxford.asp

**Table 2:** Genotype distributions of CALM1 polymorphisms in the included studies

Author & year	SOC	Ethnicity	Allele		Case			Control			Association with OA
			1	0	11	10	00	11	10	00	
**rs12885713 (promotor)**											
Mishra, 2017	NA	Caucasian	T	C	158	240	102	142	233	125	T increased/allele model (in women)
Shi, 2008	HB	Asian			9	57	117	8	70	132	Unrelated
Poulou, 2008	HB	Caucasian			38	80	36	37	103	46	Unrelated
Loughlin, 2006	NA	Caucasian			296	478	146	245	381	126	Unrelated
Mototani, 2005	HB	Asian			46	128	160	22	154	199	TT increased/recessive model
**rs2300496 (intron)**											
Mishra, 2017	NA	Caucasian	C	A	132	221	147	113	220	167	Unrelated
Mototani, 2005	HB	Asian			46	129	159	23	155	197	CC increased/recessive model
**rs3213718 (intron)**											
Valdes, 2007			T	C	T vs. C, OR & 95% CI, 0.87 (0.74, 1.03)						Unrelated
Mototani, 2005	HB	Asian			65	163	198	79	435	492	TT increased/recessive model
**rs3814843 (3′-UTR)**											
Mishra, 2017	NA	Caucasian	G	T	0	56	444	0	23	477	GG increased/recessive model
**rs2300500 (intron)**											
Mototani, 2005	HB	Asian	G	C	47	128	159	23	156	196	GG increased/recessive model
**rs3179089 (3′-UTR)**											
Mototani, 2005	HB	Asian	G	C	45	131	158	20	160	195	GG increased/recessive model

Abbreviations: HB, hospital-based; NA, not available; PB, population-based.

### Association between rs12885713 polymorphism and OA susceptibility

**Figure 2:**
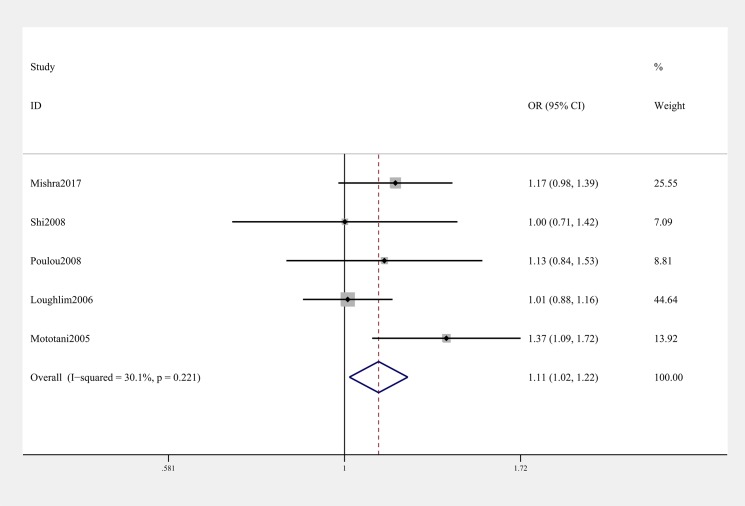
Forest plot showing OR for the associations between the rs12885713 polymorphism and OA risk (T vs. C)

**Figure 3:**
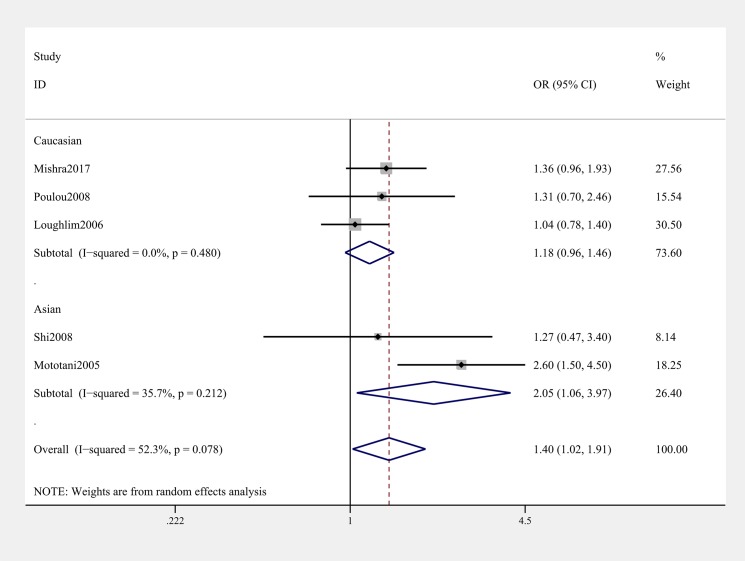
Stratification analysis by ethnicity showing OR for the association between the rs12885713 polymorphism and OA risk (TT vs. CC)

**Figure 4:**
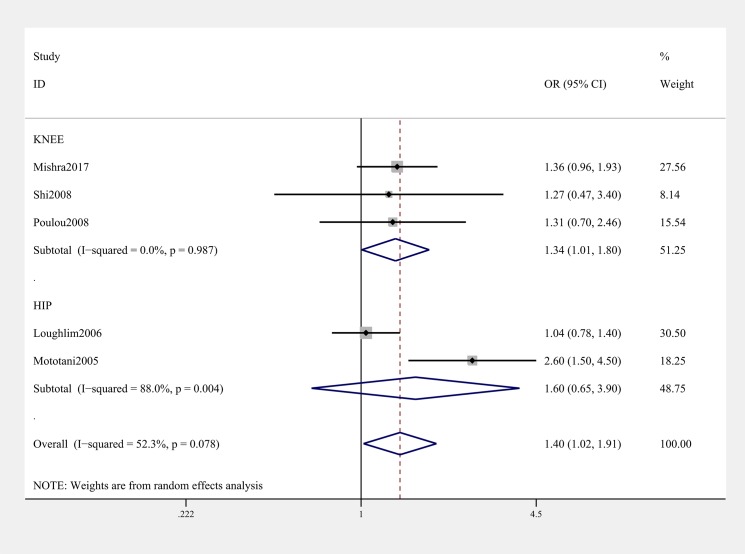
Stratification analysis by type of OA showing OR for the association between the rs12885713 polymorphism and OA risk (TT vs. CC)

General analysis showed rs12885713 polymorphism of *CALM1* gene increased OA risk (OR & 95% CI: 1.11 [1.02–1.22] in T vs. C; 1.40 [1.02–1.91] in TT vs. CC, Table 3 & Figure 2). Stratification analysis by ethnicity showed that the rs12885713 polymorphism increased the risk of OA among Asians (OR & 95% CI: 1.24 [1.03–1.51] in T vs. C; OR & 95% CI: 2.21 [1.39–3.50] in TT vs. TC + CC; 2.05 [1.06–3.97] in TT vs. CC, Table 3 and Figure 3). Subgroup analysis by OA type revealed that the rs12885713 polymorphism increased the risk of knee OA (OR & 95% CI: 1.34 [1.01, 1.80] in TT vs. CC, Table 3 and Figure 4), but not hip OA.

**Table 3:** Meta-analysis of the association between CALM1 polymorphisms and OA risk

SNP	Comparison	Category	Category	Studies	OR (95% CI)	*P*-value	*P* for heterogeneity
rs12885713	T vs. C	Total (Fixed model)		5	**1.11 (1.02–1.22)**	0.022	0.221
	Allele model	Ethnicity	Asian	2	**1.24 (1.03–1.51)**	0.024	0.140
			Caucasian	3	1.08 (0.97–1.19)	0.165	0.410
		OA type	Knee	3	1.13 (0.99–1.30)	0.079	0.735
			Hip	2	1.10 (0.97–1.23)	0.131	0.026
		Genotype method	TaqMan	2	**1.24 (1.08–1.43)**	0.002	0.286
			PCR-RFLP	3	1.03 (0.91–1.16)	0.665	0.792
	TT + TC vs. CC	Total (Fixed model)		5	1.14 (0.99–1.32)	0.071	0.732
	Dominant model	Ethnicity	Asian	2	1.13 (0.89–1.43)	0.324	0.327
			Caucasian	3	1.15 (0.96–1.38)	0.129	0.594
		OA type	Knee	3	1.15 (0.93–1.43)	0.201	0.470
			Hip	2	1.14 (0.93–1.38)	0.202	0.480
		Genotype method	TaqMan	2	**1.26 (1.03–1.56)**	0.028	0.793
			PCR-RFLP	3	1.04 (0.85–1.27)	0.699	0.895
	TT vs. TC + CC	Total (Random model)		5	1.30 (0.96–1.76)	0.086	0.023
	Recessive model	Ethnicity	Asian	5	**2.21 (1.39–3.50)**	0.001	0.234
			Caucasian	2	1.07 (0.91–1.25)	0.410	0.430
		OA type	Knee	3	1.20 (0.95–1.52)	0.120	0.902
			Hip	2	1.54 (0.60–3.93)	0.371	0.001
		Genotype method	TaqMan	2	1.67 (0.77–3.61)	0.192	0.010
			PCR-RFLP	3	1.03 (0.86–1.24)	0.742	0.514
	TT vs. CC	Total (Random model)		5	**1.40 (1.02–1.91)**	0.037	0.078
	Homozygote model	Ethnicity	Asian	2	**2.05 (1.06–3.97)**	0.033	0.212
			Caucasian	3	1.18 (0.96–1.46)	0.120	0.480
		OA type	Knee	3	**1.34 (1.01–1.80)**	0.045	0.987
			Hip	2	1.60 (0.65–3.90)	0.305	0.004
		Genotype method	TaqMan	2	1.82 (0.97–3.40)	0.063	0.051
			PCR-RFLP	3	1.10 (0.85–1.42)	0.475	0.774
	TC vs. CC	Total (Fixed model)		5	**1.08 (0.93–1.26)**	0.332	0.800
	Heterozygote model	Ethnicity	Asian	2	**0.99 (0.77–1.28)**	0.951	0.663
			Caucasian	3	1.13 (0.93–1.37)	0.206	0.672
		OA type	Knee	3	1.10 (0.88–1.38)	0.411	0.462
			Hip	2	1.06 (0.86–1.30)	0.572	0.827
		Genotype method	TaqMan	2	1.14 (0.91–1.43)	0.246	0.380
			PCR-RFLP	3	1.03 (0.83–1.27)	0.813	0.811
rs2300496	C vs. A	Total (Fixed model)		2	**1.23 (1.07–1.42)**	0.003	0.329
	Allele model						
	CC + CA vs. AA dominant model	Total (Fixed model)		2	1.21 (0.99–1.48)	0.059	0.955
	CC vs. CA + AA	Total (Random model)		2	1.67 (0.85–3.27)	0.133	0.024
	Recessive model						
	CC vs. AA	Total (Random model)		2	1.75 (0.95–3.21)	0.072	0.055
	Homozygote model						
	CA vs. CC	Total (Fixed model)		2	1.09 (0.88–1.35)	0.432	0.641
	Heterozygote model						
rs3213718	T vs. C	Total (Fixed model)			1.09 (0.98-1.21)	0.105	0.153
	Allele model						

*Bold values are statistically significant (*P*<0.05).

**Figure 5:**
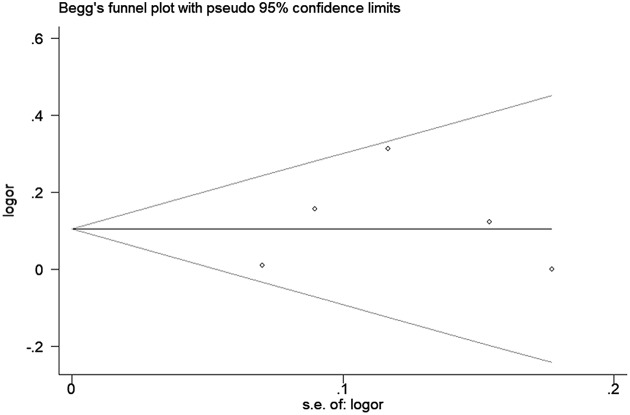
Begg’s tests between the rs12885713 polymorphism and OA (T vs. C)

Sensitivity analysis was used to determine the pooled ORs regarding the effects of this SNP on OA risk; the results indicated that our data were stable and credible. The results of false-positive test also proved our findings (Supplementary Table S2). Neither Egger’s nor Begg’s tests revealed obvious publication bias for the rs12885713 polymorphism (Figure 5).

### Association between rs2300496/rs3213718 polymorphisms and OA susceptibility

The results of pooled analysis on the association between *CALM1* gene rs2300496/ rs3213718 polymorphisms and OA risk are shown in Table 3. The C allele (OR & 95% CI: 1.23 [1.07–1.42] in C vs. A) for the rs2300496 polymorphism increased the risk of OA as verified by a false-positive test (Supplementary Table S2), whereas the association was not found under the other four genetic models. Thus, we hypothesized that the rs2300496 polymorphism of CALM1 gene may increase the risk of OA susceptibility.

Owing to the incomplete genotype distribution data reported by Valdes et al. [22], only an allele model was used for the rs3213718 polymorphism, which indicated no association. Stratification analysis was not performed, owing to data unavailability.

The rs3814843, rs2300500, and rs3179089 polymorphisms of the *CALM1* gene were investigated in only one study [17,19], which reported significant associations (Table 2). Nevertheless, further replication studies are required to confirm the associations.

### Functional predictions

**Figure 6:**
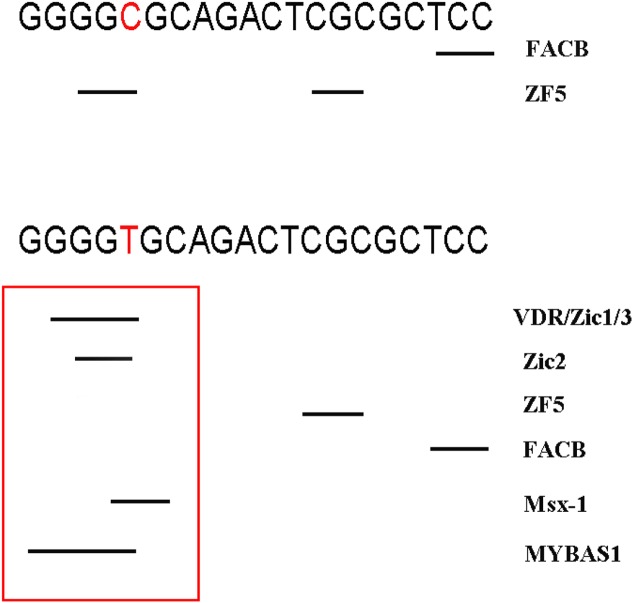
Changed TFBS of rs12885713

Rs12885713 was located in TFBS. As shown in Figure 6 for rs12885713, if nucleotide C was changed to T, four new TFBS of the VDR, Zic1, Zic3, and MYBAS appeared, thus indicating that polymorphisms of this site may change the transcriptional efficiency of *CALM1*.

Two SNPs (rs3814843 and rs3179089) in the 3′-UTR of CALM1 may affect binding to microRNA. According to the MirSNP database (http://bioinfo.bjmu.edu.cn/mirsnp/search/), the CALM1 gene binds has-miR-497-5p when the nucleotide changes from A to C (for rs3814843). Has-miR-497-5p is a negative regulator of SMAD3 gene expression [36], and SMAD3 represses MMP13 expression, thereby maintaining articular cartilage and preventing OA [37]. We hypothesized that the minor allele of rs3814843 conferred susceptibility to OA by binding to has-miR-497-5p. For rs3179089, allelic changes affect binding to microRNAs, but these microRNAs have not been reported in the literature (Supplementary Figure S1).

## Discussion

To our knowledge, this is the first meta-analysis to investigate the associations between 6 *CALM1* gene SNPs and OA risk. Although the definite pathogenesis of OA remains unclear, genetic factors are considered to be strong determinants. Ca^2+^-CALM signaling has a known role in cartilage phenotype maintenance and chondrogenesis, and microarray analysis [17] has shown that CALM1 expression is elevated in both hip and knee OAs. Many studies [17–22] have explored the association between *CALM1* gene SNPs and OA susceptibility. Mototani et al. [17] have found that the rs12885713 polymorphism in the core promoter region of CALM1 is associated with hip OA in a Japanese population and CALM1 expression is elevated in cultured chondrocytes and articular cartilage. Loughlin et al. [18] have suggested that the rs12885713 polymorphism is not a risk factor for OA in the U.K., a result confirmed by another Caucasian study from Greece [20]. A Chinese study [21] also did not find a significant association for this SNP, results similar to findings among Indians. However, Mishra et al. [19] have indicated that *CALM1* gene rs12885713 polymorphism increases the risk of OA in females. The findings are clearly conflicting between Asians and Caucasians, possibly for the following six reasons. First, the inclusion criteria differed among studies. For example, Mototani et al. [17] used clinical symptoms and radiological evidence of joints, whereas Loughlin et al. [18] enrolled OA cases who underwent elective hip joint replacement. Second, the allele frequencies of the rs12885713 polymorphism in the cases were diverse. Third, the clinical phenotypes of OA were different. Fourth, the affected joint sites differed. Fifth, the genetic background of OA may vary among races. Finally, the sample sizes may also account.

Owing to their limited sample sizes, previous single studies may have been underpowered and thus have presented conflicting findings, especially given the diverse inheritance of the heterogeneous and complex OA etiology, different ethnicities, clinical heterogeneity, and other causes. Therefore, we conducted this meta-analysis. Our data showed that *CALM1* gene rs12885713 polymorphism increases the risk of OA. Furthermore, stratification analyses by ethnicity and OA types indicated that this polymorphism increases the risk of OA among Asians and in the knee, respectively. There are several possible reasons for the different findings regarding the rs12885713 polymorphism between Caucasians and Asians. First, genetic heterogeneity for OA exists in different populations. Second, these discrepancies may be explained by clinical heterogeneity. Third, the sample sizes of the Asian populations were not large enough relative to Caucasian populations to support a clear conclusion. Additionally, the different OA types (knee and hip OA) and varying clinical parameters of different populations may also be potential reasons for the inconclusive findings. As for the conflicting findings according to the OA site, we believe that *CALM1* gene rs12885713 polymorphism may be a specific variant for knee OA but not hip OA. Furthermore, the different characteristics of the OA groups (such as age and sex) and disease severity may also be possible reasons for the discrepancies. Finally, the varying environmental factors may also have contributed, because the interaction between genetic factors and environmental factors can eventually lead to the development of OA. Functional predictions indicated that the rs12885713 polymorphism may change the transcriptional efficiency of the *CALM1* gene. Thus, we assumed that changes in the transcriptional function by rs12885713 polymorphism may eventually alter CALM1 protein translation, thereby increasing the risk of OA. In addition, we believe that the effect of the rs12885713 polymorphism may be subtle, because this SNPs may be in linkage disequilibrium with other variants affecting the risk of OA (Supplementary Figure S2). Thus, further studies investigating other SNPs are urgently needed. Our meta-analysis also showed that the *CALM1* gene rs2300496 polymorphism, but not the rs3213718 polymorphism, may increase the risk of OA. As for other SNPs of the *CALM1* gene, the rs3814843, rs2300500, and rs3179089 polymorphisms were significantly associated with OA risk, although further replication studies are needed to confirm whether these SNPs influence the genetic risk of OA.

Some limitations of this meta-analysis should be considered. First, owing to limited data, we were unable to conduct stratification analyses of other potential factors, such as age, sex, and OA onset age. Second, our results were based on unadjusted estimates of confounding factors, which might have affected the final results. Third, although funnel plots and Egger’s tests revealed no publication bias, selection bias could not be fully avoided, because only studies published in English were searched. Fourth, we were unable to assess potential gene–gene or gene–environment interactions because of the lack of relevant data. Fifth, the conclusions of some stratification analyses of the rs12885713 polymorphism should be interpreted with caution, owing to limited sample sizes. Sixth, clinical cases should be investigated in further studies to support these analytical results. Seventh, we can only infer but cannot conclude that the *CALM1* gene rs12885713 polymorphism is a susceptibility locus for other types of OA, thus necessitating further investigation into more types of OA. Finally, five genetic models of inheritance were used; thus, type I error may have arisen through a lack of correction for multiple testing.

In conclusion, the present meta-analysis demonstrates that the rs12885713 and rs2300496 polymorphisms of the *CALM1* gene, but not the rs3213718 polymorphism, may increase the risk of OA. Owing to the study limitations, further well-designed prospective studies with large sample sizes should be performed to confirm these findings.

## Supporting information

**Supplementary Figure 1:** Changed transcription factor binding sites (TFBS) of rs3814843 and rs3179089.

**Supplemental Table:** PRISMA 2009 Checklist

**Supplementary file 2:** False-positive report probability values for associations between the CALM1 gene polymorphisms and OA risk.

## Abbreviations

CALM: calmodulin
CI: confidence interval
FPRP: false-positive report probability
HWE: Hardy–Weinberg equilibrium
NOS: Newcastle–Ottawa Scale
OA: osteoarthritis
OR: odds ratio
SNP: single-nucleotide polymorphism
SOC: source of control
TFBS: transcription factor binding sites
VDR: vitamin D receptor
