# Supplementary material for: Association between the polymorphisms of CALM1 gene and osteoarthritis risk: a meta-analysis based on observational studies
Source: Biosci Rep. 2018 Oct 31;38(5):BSR20181128. doi: 10.1042/BSR20181128 (PMC6209589; doi:10.1042/BSR20181128)
Supplement: Supplementary file 1 [file bsr20181128_Supp1.pdf]

<table>
<tr><th>Gene</th><th>miRNA</th><th>Snp</th><th>mirSVR</th><th>HCB</th><th>Effect</th><th>Allele</th><th>Score</th><th>Energy</th><th>Conservation</th><th>Start</th><th>End</th><th>binding</th></tr>
<tr><td rowspan="2">CALM1</td><td rowspan="2">hsa-miR-1324</td><td rowspan="2">rs3179089</td><td rowspan="2">-0.008</td><td rowspan="2">0.256</td><td rowspan="2">break</td><td>C</td><td>159.00</td><td>-22.35</td><td>0.374</td><td>10</td><td>33</td><td>Query: 3' cuuucACGUAUCUUAAGACAGACc 5'
|| ||:|| |||||||
Ref: 5' ttgtaTGGCCAGGATCCTGTCTGg 3'</td></tr>
<tr><td>G</td><td></td><td></td><td></td><td></td><td></td><td></td></tr>
<tr><td rowspan="2">CALM1</td><td rowspan="2">hsa-miR-218-1-3p</td><td rowspan="2">rs3179089</td><td rowspan="2">-0.019</td><td rowspan="2">0.256</td><td rowspan="2">enhance</td><td>C</td><td>149.00</td><td>-13.65</td><td>0.001</td><td>21</td><td>40</td><td>Query: 3' ggUACCACGAACUGCCUUGGUa 5'
|| ||: || |||||||
Ref: 5' ggATCCTGTCTG--GGAACCAg 3'</td></tr>
<tr><td>G</td><td>157.00</td><td>-16.25</td><td>0.001</td><td>21</td><td>40</td><td>Query: 3' ggUACCACGAACUGCCUUGGUa 5'
|| |||: || |||||||
Ref: 5' ggATCGTGTCTG--GGAACCAg 3'</td></tr>
<tr><td rowspan="2">CALM1</td><td rowspan="2">hsa-miR-4524b-3p</td><td rowspan="2">rs3179089</td><td rowspan="2"></td><td rowspan="2">0.256</td><td rowspan="2">break</td><td>C</td><td>154.00</td><td>-16.04</td><td>0.503</td><td>13</td><td>32</td><td>Query: 3' aucgucGUACUUGGACAGAg 5'
|| || |||||||
Ref: 5' tatggcCAGGATCCTGTCTg 3'</td></tr>
<tr><td>G</td><td></td><td></td><td></td><td></td><td></td><td></td></tr>
<tr><td rowspan="2">CALM1</td><td rowspan="2">hsa-miR-4661-3p</td><td rowspan="2">rs3179089</td><td rowspan="2"></td><td rowspan="2">0.256</td><td rowspan="2">break</td><td>C</td><td>140.00</td><td>-17.95</td><td>0.605</td><td>7</td><td>28</td><td>Query: 3' accugaucgagacaCCUAGGAc 5'
|||||||
Ref: 5' ttcttgtatggccaGGATCCTg 3'</td></tr>
<tr><td>-</td><td></td><td></td><td></td><td></td><td></td><td></td></tr>
<tr><td rowspan="2">CALM1</td><td rowspan="2">hsa-miR-497-5p</td><td rowspan="2">rs3814843</td><td rowspan="2"></td><td rowspan="2">0.116</td><td rowspan="2">create</td><td>A</td><td></td><td></td><td></td><td></td><td></td><td></td></tr>
<tr><td>C</td><td>154.00</td><td>-25.67</td><td>0.000</td><td>8</td><td>29</td><td>Query: 3' ugUUUGGU-GUCACACGACGAc 5'
| |:|: | |||||||||
Ref: 5' agACATCGCCCATGTGCTGCTg 3'</td></tr>
</table>

Supplementary Figure 1: Changed transcription factor binding sites (TFBS) of rs3814843 and rs3179089.

Query SNP: rs12885713 and variants with $r^2 >= 0.8$

chr	pos (hg38)	LD (r^2)	LD (D')	variant	Ref	Alt	AFR freq	AMR freq	ASN freq	EUR freq	SiPhy cons	Promoter histone marks	Enhancer histone marks	DNAse	Proteins bound	Motifs changed	NHGRI/EBI GWAS hits	GRASP QTL hits	Selected eQTL hits	GENCODE genes	dbSNP func annot
14	90386785	0.96	0.99	rs34595809	CG	C	0.07	0.40	0.21	0.58			5 tissues			8 altered motifs				5.5kb 3' of AL512791.1	
14	90392032	0.96	0.99	rs7150417	C	T	0.09	0.39	0.22	0.59						11 altered motifs				4.5kb 5' of CALM1	intronic
14	90397013	1	1	rs12885713	C	T	0.08	0.40	0.22	0.59		24 tissues	BLD	53 tissues	55 bound proteins	NRSF,TR4			1 hit	CALM1	5'-UTR
14	90398504	0.99	0.99	rs2300496	C	A	0.06	0.40	0.22	0.59		24 tissues		47 tissues	9 bound proteins	7 altered motifs			3 hits	CALM1	intronic
14	90399373	0.98	0.99	rs2300500	G	C	0.10	0.40	0.22	0.59		20 tissues	9 tissues	16 tissues		Pou5f1			3 hits	CALM1	intronic
14	90403569	0.87	0.99	rs3213718	C	T	0.38	0.45	0.23	0.62			6 tissues	MUS,BLD,SKIN		5 altered motifs		1 hit	2 hits	RP11-471B22.2	intronic
14	90407068	0.9	0.97	rs3179089	C	G	0.06	0.38	0.22	0.58			10 tissues	6 tissues		Smad3,Smad			3 hits	CALM1	3'-UTR

PRISMA 2009 Checklist

Section/topic	#	Checklist item	Reported on page #
**TITLE**			
Title	1	Identify the report as a systematic review, meta-analysis, or both.	1
**ABSTRACT**			
Structured summary	2	Provide a structured summary including, as applicable: background; objectives; data sources; study eligibility criteria, participants, and interventions; study appraisal and synthesis methods; results; limitations; conclusions and implications of key findings; systematic review registration number.	2
**INTRODUCTION**			
Rationale	3	Describe the rationale for the review in the context of what is already known.	3-4
Objectives	4	Provide an explicit statement of questions being addressed with reference to participants, interventions, comparisons, outcomes, and study design (PICOS).	3-4
**METHODS**			
Protocol and registration	5	Indicate if a review protocol exists, if and where it can be accessed (e.g., Web address), and, if available, provide registration information including registration number.	4
Eligibility criteria	6	Specify study characteristics (e.g., PICOS, length of follow-up) and report characteristics (e.g., years considered, language, publication status) used as criteria for eligibility, giving rationale.	5
Information sources	7	Describe all information sources (e.g., databases with dates of coverage, contact with study authors to identify additional studies) in the search and date last searched.	4-5
Search	8	Present full electronic search strategy for at least one database, including any limits used, such that it could be repeated.	4-5
Study selection	9	State the process for selecting studies (i.e., screening, eligibility, included in systematic review, and, if applicable, included in the meta-analysis).	5
Data collection process	10	Describe method of data extraction from reports (e.g., piloted forms, independently, in duplicate) and any processes for obtaining and confirming data from investigators.	5-6
Data items	11	List and define all variables for which data were sought (e.g., PICOS, funding sources) and any assumptions and simplifications made.	5-6
Risk of bias in individual studies	12	Describe methods used for assessing risk of bias of individual studies (including specification of whether this was done at the study or outcome level), and how this information is to be used in any data synthesis.	6
Summary measures	13	State the principal summary measures (e.g., risk ratio, difference in means).	6
Synthesis of results	14	Describe the methods of handling data and combining results of studies, if done, including measures of consistency (e.g., I^2) for each meta-analysis.	6

PRISMA 2009 Checklist

Section/topic	#	Checklist item	Reported on page #
Risk of bias across studies	15	Specify any assessment of risk of bias that may affect the cumulative evidence (e.g., publication bias, selective reporting within studies).	--
Additional analyses	16	Describe methods of additional analyses (e.g., sensitivity or subgroup analyses, meta-regression), if done, indicating which were pre-specified.	6
**RESULTS**			
Study selection	17	Give numbers of studies screened, assessed for eligibility, and included in the review, with reasons for exclusions at each stage, ideally with a flow diagram.	7
Study characteristics	18	For each study, present characteristics for which data were extracted (e.g., study size, PICOS, follow-up period) and provide the citations.	7
Risk of bias within studies	19	Present data on risk of bias of each study and, if available, any outcome level assessment (see item 12).	8
Results of individual studies	20	For all outcomes considered (benefits or harms), present, for each study: (a) simple summary data for each intervention group (b) effect estimates and confidence intervals, ideally with a forest plot.	7
Synthesis of results	21	Present results of each meta-analysis done, including confidence intervals and measures of consistency.	7-8
Risk of bias across studies	22	Present results of any assessment of risk of bias across studies (see Item 15).	8
Additional analysis	23	Give results of additional analyses, if done (e.g., sensitivity or subgroup analyses, meta-regression [see Item 16]).	8-9
**DISCUSSION**			
Summary of evidence	24	Summarize the main findings including the strength of evidence for each main outcome; consider their relevance to key groups (e.g., healthcare providers, users, and policy makers).	9-11
Limitations	25	Discuss limitations at study and outcome level (e.g., risk of bias), and at review-level (e.g., incomplete retrieval of identified research, reporting bias).	11
Conclusions	26	Provide a general interpretation of the results in the context of other evidence, and implications for future research.	12
**FUNDING**			
Funding	27	Describe sources of funding for the systematic review and other support (e.g., supply of data); role of funders for the systematic review.	--

From: Moher D, Liberati A, Tetzlaff J, Altman DG, The PRISMA Group (2009). Preferred Reporting Items for Systematic Reviews and Meta-Analyses: The PRISMA Statement. PLoS Med 6(7): e1000097. doi:10.1371/journal.pmed1000097

For more information, visit: www.prisma-statement.org.

Supplementary file 2 False-positive report probability values for associations between the CALM1 gene polymorphisms and OA risk.

Variables	OR(95%CI)	*P* value	Prior Probability				
			0.25	0.1	0.01	0.001	0.0001
rs12885713 T vs. C							
Total	1.11(1.02,1.22)	0.022	**0.128**	0.306	0.829	0.980	0.998
Asians	1.24(1.03, 1.51)	0.024	**0.137**	0.323	0.840	0.981	0.998
rs12885713 TT vs. CC							
Total	1.40(1.02, 1.91)	0.037	**0.177**	0.393	0.877	0.986	0.999
Asian	2.05(1.06, 3.97)	0.033	**0.166**	0.373	0.868	0.985	0.998
Knee OA	1.34(1.01, 1.80)	0.045	**0.186**	0.407	0.883	0.987	0.999
rs2300496 C vs. A							
Total	1.23(1.07, 1.42)	0.003	**0.020**	0.057	0.401	0.871	0.985
